# Anti-proliferative Activity and Apoptosis Induction of Extracts and Fractions of *Stachys lavandulifolia* on Lung (H1299), Ovarian (A2780) and Breast (MCF-7) Human Cancer Cell Lines

**DOI:** 10.5812/ijpr-152370

**Published:** 2025-08-26

**Authors:** Leila Hosseinzadeh, Pouria Hajmomeni, Mohadeseh Salehi, Masuod Modarresi, Fereshteh Jalilian

**Affiliations:** 1 Pharmaceutical Sciences Research Center, Faculty of Pharmacy, Kermanshah University of Medical Sciences, Kermanshah, Iran; 2 Student Research Committee, Kermanshah University of Medical Sciences, Kermanshah, Iran

**Keywords:** *Stachys lavandulifolia*, Herbal Medicine, Toxicity, Extracts

## Abstract

**Background:**

At present, regarding the optimal potential for prevention and treatment of cancer, medicinal plants have received more attention than before. *Stachys lavandulifolia* is one of the 34 *Stachys* species found in various regions such as Iran. Although effective secondary metabolites have been identified in several species of the *Stachys* genus, research on the anticancer effects of extracts of *S. lavandulifolia* is limited.

**Methods:**

In the present study, different extracts of *S. lavandulifolia *(n-hexane, chloroform, ethyl acetate, ethanol, hydroethanol, and water) were acquired, and three human cancer cell lines (H1299, MCF-7, and A2780) were used to analyze their anticancer effects.

**Results:**

The chloroform and ethyl acetate extracts provided the highest toxicity against A2780 and were fractioned into A-E and F-J, respectively. Fractions C and F resulted in reducing the mitochondrial membrane potential (MMP), while fractions C, E, F, and I led to an increase in the activity of the caspase-3 enzyme. Fractions B, C, and I increased the reactive oxygen species (ROS) in the A2780 cells.

**Conclusions:**

Considering the reduction in MMP and the increase in caspase-9 by fraction C, it can be said that apoptosis is induced by the intrinsic pathway.

## 1. Background

Cancer is a group of diseases characterized by the uncontrolled growth and spread of abnormal cells. It is becoming a leading cause of death worldwide, and the incidence of the disease is estimated to grow every year (1). Around 14.1 million new cases are diagnosed annually (including non-melanoma skin cancer), causing the death of 8.8 million people (15.7% of mortality) worldwide (2). Female breast cancer has surpassed lung cancer as the most commonly diagnosed cancer, with an estimated 2.3 million new cases (11.7%), followed by lung (11.4%) and colorectal (10.0%) cancer. Ovarian cancer is also among the most commonly diagnosed cancers among women globally, with approximately 239,000 new cases and 152,000 deaths reported each year (3).

Currently, there is growing interest in medicinal plants for cancer prevention and treatment. This interest is primarily due to their good therapeutic efficacy, low side effects, and lower cost compared to synthetic products (4, 5). Evidence suggests that research on medicinal plants, in addition to using their natural compounds for treatment, could lead to the synthesis of certain chemical drugs based on their natural active ingredients. For centuries, plants and their extracts have been widely used in traditional medicine to treat various diseases (6).

A subset of these plants belongs to the *Stachys* genus. *Stachys lavandulifolia* is one of the 34 *Stachys* species found in different regions such as Iran, Turkey, and Iraq (7). Research has indicated that the extract of this plant exhibits an anxiolytic effect with relatively lower sedative properties when compared to diazepam (8). Several secondary metabolites such as iridoids, flavonoids, diterpenoids, and phenolic acids have been identified in various species of the *Stachys* genus (9). Monoterpenoids and sesquiterpenoids can be identified in the essential oils of *Stachys* species. The dominant volatile compounds observed in the genus include linalool, germacrene D, caryophyllene, as well as α- and β-pinene (10). Studies have documented the presence of lavandulifolioside A, lavandulifolioside B, verbascoside, and leucosceptoside A in the polar fractions of *S. lavandulifolia* (11).

Previous studies have also identified the presence of several cytotoxic compounds in S*. lavandulifolia*. Notably, Delnavazi et al. investigated the aerial parts of *S. lavandulifolia* and isolated methoxylated flavonoids, including chrysosplenetin, kumatakenin, and viscosine, as key constituents with significant cytotoxic activity. The presence of these polymethoxylated flavonoids is suggested to underlie the plant’s cytotoxic potential, possibly due to their ability to interfere with cellular proliferation pathways (12). Other studies on the genus *Stachys* have reported flavonoids, phenolic acids, and terpenoids as common secondary metabolites with cytotoxic properties, suggesting that *S. lavandulifolia* may share similar phytochemical profiles contributing to its bioactivity (13).

## 2. Objectives

In the present study, we aimed to evaluate and compare the cytotoxic and apoptotic effects of different extracts of the aerial parts of *S. lavandulifolia* on the lung (H1299), ovarian (A2780), and breast (MCF-7) human cancer cell lines. Additionally, we sought to identify the most potent fractions responsible for the plant’s cytotoxic activity and investigate their potential mechanisms of action.

## 3. Methods

### 3.1. Reagents and Materials

Triton^®^ X-100, 3-(4,5-dimethylthiazol-2-yl)-2,5-diphenyltetrazolium bromide (MTT), rhodamine 123 fluorescent dye, fluorescent probe 2,7-dichlorofluorescein diacetate (DCF-DA), caspase-3 & 9 detection kits, Bradford reagent, and dimethyl sulfoxide (DMSO) were purchased from Sigma Aldrich (St. Louis, MO, USA). Dulbecco’s modified Eagle’s medium-F12 (DMEM-F12), fetal bovine serum (FBS), and penicillin/streptomycin were obtained from Bio-Idea (Iran). Trypsin-EDTA was prepared from Bon Yakhteh (Iran). Other chemicals and all the solvents used for extraction and fractionation were purchased from Merck (Germany) and Dr. Mojallali (Iran).

### 3.2. Preparation, Authentication, and Extraction of Stachys lavandulifolia

In late May, the *S. lavandulifolia* plant was collected from the mountain hillsides of Bostanabad county, East Azerbaijan, Iran. Verification and identification were conducted by Dr. Nasrin Jalilian from the Research Center of Agriculture and Natural Resources of Kermanshah province, and a specimen (No. 5594) was deposited at the Herbarium. Six different extracts (n-hexane, chloroform, ethyl acetate, ethanol, hydroethanol, and water) were obtained using the maceration method. Briefly, 30 g of dried aerial parts of the *S. lavandulifolia* (leaves, stems, and flowers) were macerated separately with 1500 mL of the mentioned solvents (in a 1:50 ratio). After maceration, the extracts were filtered and centrifuged at 3000 rpm for 30 minutes to remove any solid particles. The extracts were then dried using a rotary evaporator at a temperature below 40°C. To prevent the degradation of secondary metabolites, the dried extracts were stored at -20°C until cytotoxicity assessments were performed.

### 3.3. Fractionation of Chloroform Extract

The crude extract that was acquired underwent solid-phase extraction (SPE) utilizing a silica gel cartridge. The SPE tube was packed with 10 g of silica gel (230 - 400 mesh) after activation in the oven at a temperature of 120°C for 1 hour. After equilibration with pure chloroform, fractionation was performed. For this purpose, 1.68 g of total chloroform extract was superficially adsorbed onto 3.36 g of silica and loaded on 10 g of silica gel inside the SPE tube. The system was equipped with a vacuum pump and Buchner flask, and 100 mL of chloroform solvents 50% in n-hexane, chloroform 100%, ethyl acetate 10% in chloroform, ethyl acetate 100%, and methanol 100% were passed through the SPE tube, respectively. The mentioned solvents were separately collected after passing through the tube. Accordingly, the fractionation was carried out for the chloroform extract. Each collected fraction was dried using a rotary evaporator and labeled A to E. The net weight of each fraction was recorded.

### 3.4. Fractionation of Ethyl Acetate Extract

The same procedure was performed for the ethyl acetate extract as for the chloroform extract. A total of 1.37 g of the dried ethyl acetate extract was superficially adsorbed on 2.74 g of silica gel (230 - 400 mesh) and loaded onto 10 g of silica gel. At this stage, the chloroform solvents 50% in n-hexane, chloroform 70% in n-hexane, chloroform 100%, ethyl acetate 100%, and methanol 100% were passed through the SPE tube. The mentioned solvents were separately collected after passing through the tube. Each collected fraction was dried using a rotary evaporator and labeled F to J.

### 3.5. Preparing the Solutions from the Total Extracts and Fractions

In total, six extracts and ten fractions were obtained. After preparing the total extracts and fractions and drying them, a specific amount of a mixture of methanol and DMSO (in the ratio 1:9) was added to each, and the solutions were provided by heating, using a test tube shaker device and an ultrasonic device. After preparation of the solutions, specific amounts of them were transferred to microtubes and, after labeling, they were stored at -20°C. To evaluate the toxicity of the obtained total extracts and fractions, the stock concentrations had to be obtained from them. For this purpose, volumes of 5, 10, 25, 50, and 75 μL of the extract or the fraction were dissolved in 95, 90, 75, 50, and 25 μL DMSO, respectively.

### 3.6. Cell Culture

H1299, MCF-7, and A2780 human carcinoma cell lines were provided by the Pasteur Institute of Iran. The cells were grown in DMEM-F12 supplemented with 10% heat-inactivated FBS with 1% penicillin (100 U/mL) and streptomycin (100 μg/mL) in a humidified atmosphere containing 95% air and 5% CO_2_ at a temperature of 37°C. The medium was changed at specified time intervals until the cell proliferation reached an appropriate population density of 70 - 80% confluence.

#### 3.6.1. Evaluation of Cytotoxicity Using the 3-(4,5-Dimethylthiazol-2-yl)-2,5-Diphenyltetrazolium Bromide Method

The toxicity of the extracts and fractions was studied using the MTT assay. To evaluate cell viability, the cells were cultured in a 96-well plate with a density of 20,000 cells per well and were incubated in (DMEM-F12 supplemented with inactivated FBS (10% v/v) and 1% penicillin/streptomycin (100 U/mL: 100 U/mL) for cell growth at 37°C in a humidified incubator containing 5% CO_2_. Once cell proliferation reached 70% - 80%, they were exposed to different concentrations of extracts and fractions (0 - 100 µg/mL). The different concentrations of extracts and fractions were prepared by serial dilution in DMSO as a suitable solvent. It should be noted that the DMSO concentration should not exceed 1% in the medium, so the stock concentration was made 100 times the desired concentration. A group of cells was treated with DMSO alone as a control. After 24 hours, the medium was replaced with 20 μL of 0.5 mg/mL of MTT in medium, and plates were further incubated for 2 - 4 hours at 37°C. After that, MTT-formazan products were dissolved in 100 µL DMSO. The absorbance was measured at 490 nm using an ELISA microplate reader (BioTek Instruments, USA). Growth inhibition was calculated, and IC_50_ values were determined, corresponding to the concentration required for inhibition of growth of 50% of cells.

#### 3.6.2. Measurement of Mitochondrial Membrane Potential

One of the important factors involved in inducing apoptosis through the intrinsic pathway is mitochondrial membrane potential (MMP). In this study, MMP was assessed by rhodamine 123 as a fluorescent cationic dye. Rhodamine and its derivatives are fluorescent cationic lipophilic dyes that penetrate the cell, and the intensity of their fluorescence is indicative of the amount of change in MMP (14). For this purpose, the most sensitive cell line, A2780, was treated with the IC_50_concentration of the selected fractions in 6-well plates for 24 hours. At the end of treatment, cells were incubated with rhodamine 123 (15 μL, 20 μM) for 30 minutes at 37°C. Thereafter, the fluorescence of lysed cells by Triton^™^ X100 was measured at an excitation wavelength of 488 nm and an emission wavelength of 590 nm using a fluorescence microplate reader (BioTek, H1 M, USA). The Bradford assay was also used for the determination of protein content of each sample (15).

#### 3.6.3. Determination of Activity of Caspase-3 and -9

In this experiment, the activity of caspase-3 and -9 enzymes was studied using colorimetric caspase-3 and -9 kits (Sigma, USA). For this purpose, apoptosis was induced in the cell lines using the IC_50_ concentration of selected fractions. Briefly, after 24 hours, lysis buffer was added to cells, and then the lysed cells were centrifuged at 16,000 - 20,000g for 10 - 15 minutes at 4°C, and the supernatants were transferred to new microtubes. Next, the samples were incubated with the substrate of caspase-3 (DEVD pNA) and caspase-9 (LEHD pNA) at 37°C for 2 hours. Finally, the release of p-nitroaniline was measured using a microplate reader (BioTek, H1M) at 405 nm. The protein content was determined by the Bradford assay.

#### 3.6.4. Morphological Assessment of Apoptotic Cells by DAPI Staining

In the next stage of our experiment, to confirm the induction of apoptosis by the active fractions, the morphological changes of A2780 cells were assayed using DAPI dye (16). Briefly, the cells were incubated for 24 hours in a humidified atmosphere of 5% CO_2_ at 37°C with the IC_50_ concentration of active fractions (C, E, F, and I). At the end of the incubation, cells were stained using DAPI in PBS (2.5 μg/mL) for 10 minutes at room temperature in a dark condition. Finally, the stained cells were washed with PBS, and the nuclear morphological changes were viewed at tenfold magnification by BioTek Cytation 5 cell imaging multimode reader.

#### 3.6.5. Determination of Intracellular Reactive Oxygen Species 

The DCF-DA (Sigma-Aldrich, Germany) is used for the measurement of intracellular reactive oxygen species (ROS) levels. It is non-fluorescent and changes to fluorochrome DCF upon oxidation as it crosses the plasma membrane. At first, the cells were treated with the IC_50_ concentration of the selected fractions. After the completion of the treatment (24 hours), DCF reagent was added to the culture medium. The plates were kept in the incubator for another 45 minutes at 37°C. Thereafter, cells were lysed with Triton^™^ X100, and the fluorescence was measured at 488 nm (excitation wavelength) and 528 nm (emission wavelength) utilizing a fluorescence microplate reader (BioTek, H1 M, USA).

### 3.7. Statistical Analysis

In the present study, all the experiments were conducted in triplicate, and reported values were represented as the mean value ± SEM. Individual groups were compared with Tukey’s post hoc test or Student’s *t*-test, with a value of P < 0.05 considered statistically significant.

## 4. Results

### 4.1. The Extraction Efficiency of Stachys lavandulifolia

The extraction efficiency of the various total extracts (n-hexane, chloroform, ethyl acetate, ethanol, hydroethanol, and water) from the *S. lavandulifolia* plant is summarized in Table 1. 

**Table 1.** Extraction Efficiency for Preparing the Total Extracts from the Plant

Name of Total Extract	Extraction Efficiency (%)
**Water**	19.936
**Ethanol**	8.549
**Hydro ethanol**	19.837
**Ethyl acetate**	2.962
**Chloroform**	1.684
**n-hexane**	3.149

### 4.2. Anti-proliferative Effects of Extracts and Fractions

According to our results, chloroform and ethyl acetate extracts showed the highest cytotoxic effects against human cancer cells (Table 2). Other extracts did not show any significant cytotoxicity against the studied cell lines at the tested doses. Therefore, in the next set of our study, the most potent extracts were fractionated (Table 3). The results of this section showed that fractions C, B, and D derived from the chloroform extract could significantly inhibit the growth of A2780 cells. Regarding H1299 cells, fractions A-D were identified as the most effective ones. Fractions C and D, with IC_50_ values of 13 and 14 μg/mL, respectively, showed acceptable effects on the MCF7 cell line (Table 4). A glance at Table 4 indicates that fractions F-I derived from the ethyl acetate extract were able to significantly inhibit the growth of A2780 cells. Also, fractions H and I were found to have a significant cytotoxic effect on the MCF7 cell line. It must be noted that the fractions derived from the ethyl acetate extract inhibited the growth of H1299 cells with less intensity than A2780 cells, and their IC_50_ values against this cell line were higher compared to MCF7 and A2780 cell lines.

**Table 2.** The Calculated IC_50_ (µg/mL) of *Stachys lavandulifolia* Total Extract on Three Human

Total Extracts	A2780 (IC_50_ µg/mL)	MCF-7 (IC_50_ µg/mL)	H1299 (IC_50_ µg/mL)
**Water**	> 100	> 100	> 100
**Ethanol**	> 100	95	> 100
**Hydroethanol**	> 100	87	> 100
**Ethyl acetate**	25	28	29
**Chloroform**	11	14	31
**n-Hexane**	113	> 100	> 100

**Table 3.** Results Obtained from Solid-Phase Extraction of Total Chloroform (1.68 g) and Ethyl Acetate Extract (1.37 g)

Fraction	Name	Dried Fraction Weight (gr)
**Chloroform extract (%)**		
Chloroform 50 in n-hexane	A	0.2395
Chloroform 100	B	0.7091
Ethyl acetate 10 in chloroform	C	0.2327
Ethyl acetate 100	D	0.2004
Methanol 100	E	0.2424
**Ethyl acetate extract (%)**		
Chloroform 50 in n-hexane	F	0.4969
Chloroform 70 in n-hexane	G	0.1587
Chloroform 100	H	0.1305
Ethyl acetate 100	I	0.151
Methanol 100	J	0.2407

**Table 4.** The IC_50_ Concentration (µg/mL) of Chloroform and Ethyl Acetate Fractions on Studied Human Carcinoma Cell Lines

Cell Line	Chloroform Extract (IC_50_ µg/mL)					Ethyl Acetate Extract (IC_50_ µg/mL)				
	A	B	C	D	E	F	G	H	I	J
**MCF-7**	> 100	41	13	14	> 100	> 100	62	31	29	65
**A2780**	84	12.5	12	11	38	19	31	29	16	48.3
**H1299**	10	18	9	11	92	60	48	62.5	48	52

The cytotoxic effect of the well-known chemotherapeutic drug, doxorubicin, was also assayed in the carcinoma cell lines. Our results indicated that doxorubicin is able to inhibit the growth of A2780, MCF7, and H1299 cell lines with IC_50_ values of 2.85 µg/mL, 27.3 µg/mL, and 4.76 µg/mL, respectively. Interestingly, fractions C, D, F, and I were more potent than doxorubicin in A2780. Therefore, according to the obtained results, fractions F-I of the ethyl acetate extract and B-E from the chloroform extract were selected for the identification of mechanisms involved in apoptosis in A2780, the most sensitive cancer cell line, by evaluation of intracellular ROS, caspase-3 and -9 activities, and MMP.

### 4.3. Mitochondrial Membrane Potential Assay

**Figure 1.**
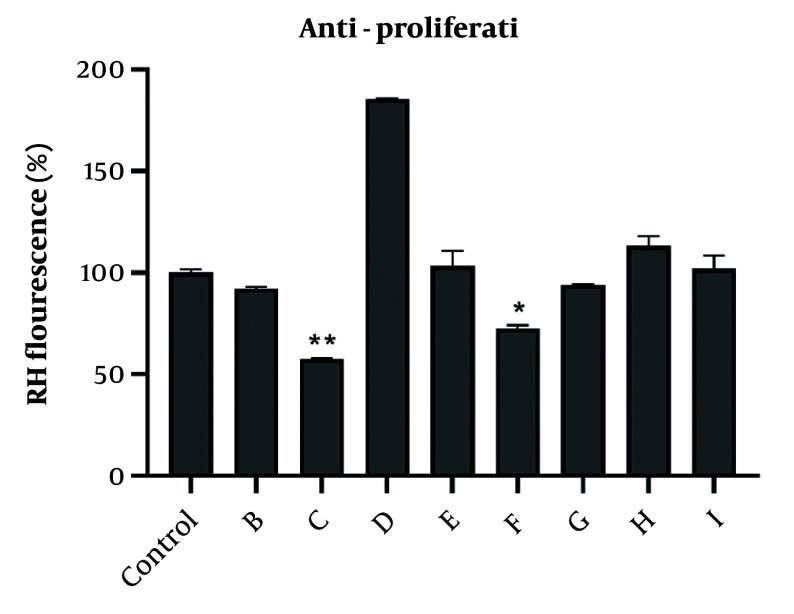
Effect of different concentrations of potent fractions obtained from total extracts of chloroform and ethyl acetate on MMP collapses detected by rhodamine 123 fluorescence. Fractions were used at IC_50_ concentration. Rhodamine 123 fluorescence intensity (%) expressed MMP in experimental groups. Data were presented as the mean ± SEM, n = 3. * P < 0.05, and ** P < 0.01 indicate significant differences compared to control group.

We used rhodamine 123 dye to evaluate the ability of potent fractions in altering MMP in A2780 cells. The obtained results showed that among the efficient fractions involved in triggering cytotoxicity, only fractions C and F could significantly reduce the MMP. Other fractions were not able to reduce MMP in A2780 cells (Figure 1). 

**Figure 2.**
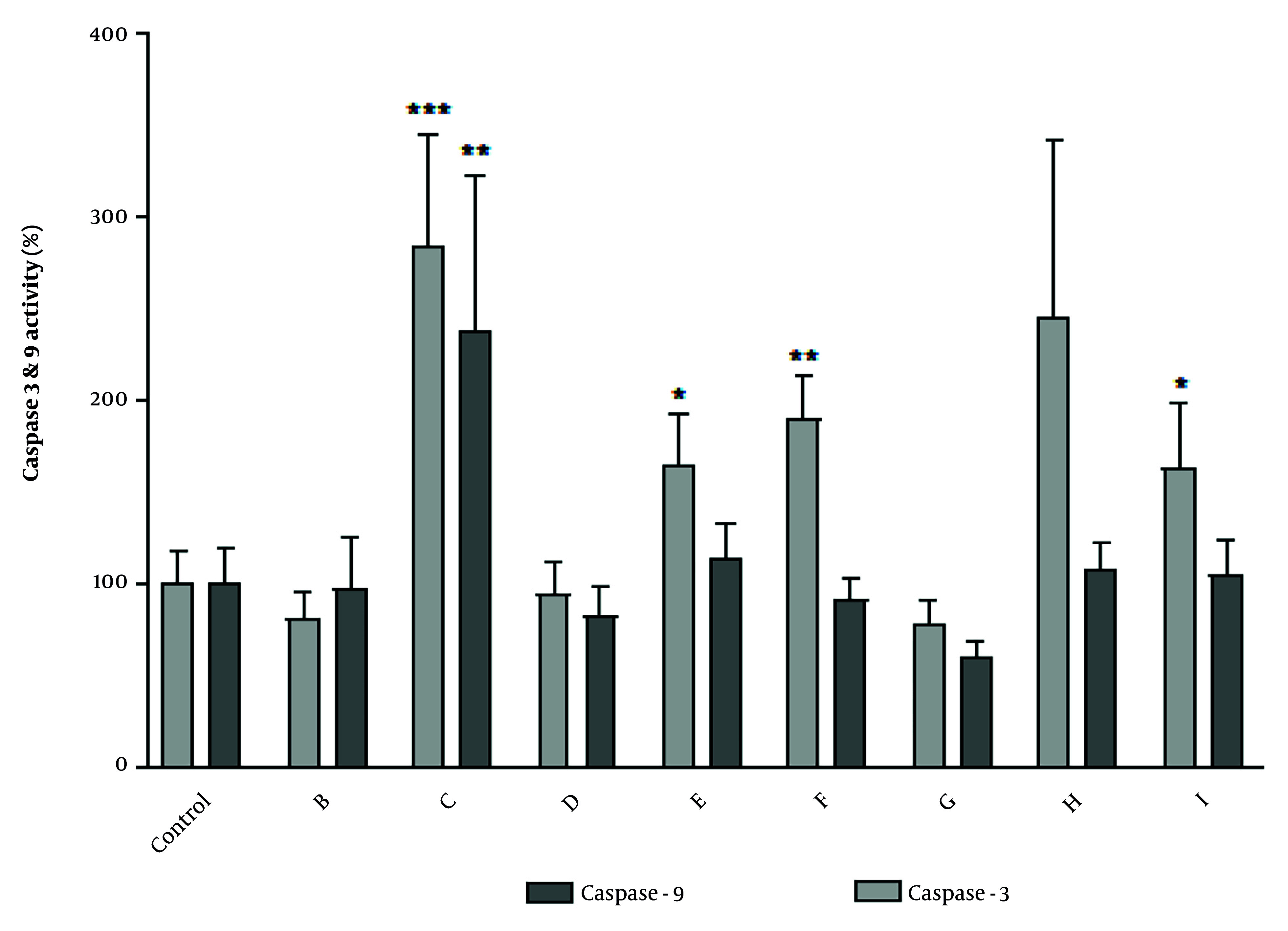
The effect of IC_50_ concentration of fractions obtained from total extracts of chloroform and ethyl acetate on the activity of caspase-3 and-9. The activity of caspase-3 and-9 was measured based on colorimetric reaction of para-nitroaniline and data were expressed as percentages. Caspase activity in A2780 human ovary carcinoma cells following treatment with IC_50_ concentrations of potent fractions obtained from total extracts of chloroform and ethyl acetate for 24 h. Data were presented as the mean ± standard deviation, n = 3. * P < 0.05, ** P < 0.01, and *** P < 0.001 indicate significant difference with control.

In this study, to characterize the type of cell death involved in cytotoxicity induced by fractions, the activity of caspases was evaluated. The obtained results showed that 24-hour treatment with the IC_50_ concentration of selected fractions C, E, F, and I increased caspase-3 activation in the A2780 cell line (Figure 2). To determine whether the mitochondrial pathway was involved in apoptosis, we evaluated the activation of caspase-9, the apical protease in the intrinsic pathway of apoptosis (17). Fraction C was able to increase both caspase-9 and caspase-3 in A2780 cells, suggesting that the intrinsic pathway is involved in apoptosis induced by this fraction. Other fractions did not cause any significant changes in caspase-3 and -9 activity in A2780 cells.

### 4.4. Detection of Apoptosis in A2780 Cells by DAPI Staining

**Figure 3.**
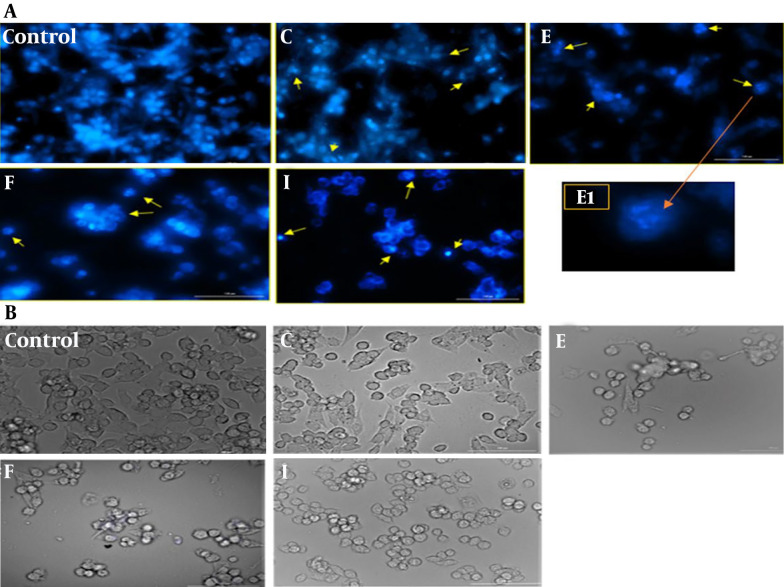
Morphological changes seen in A2780 cells after treatment with selected fractions. Cells treated with IC_50_ concentration of fractions C, E, F and I for 24 h seen A, under fluorescence; and B, light microscope following nuclear staining with DAPI. Arrow keys indicate membrane blebing, DNA fragmentation and nuclear shrinkage during apoptosis process. Scale bar: 100 µm. E1: One of the apoptotic cells that treated with IC50 concentration of fraction E shows with 40x magnification.

After 24-hour treatment with the IC_50_ concentration of selected fractions, the apoptotic alterations in cell morphology were assayed by light microscopy and fluorescence microscopy using the nuclear staining dye DAPI. As shown in Figure 3, in comparison with regular and round-shaped normal cells, treatment with fractions led to less bright blue fluorescence. Moreover, apoptotic cells, in contrast to normal cells, exhibited characteristic apoptotic changes such as membrane blebbing, cell shrinkage, chromatin condensation, and nuclear fragmentation.

### 4.5. Effect of Fractions on Reactive Oxygen Species Generation

**Figure 4.**
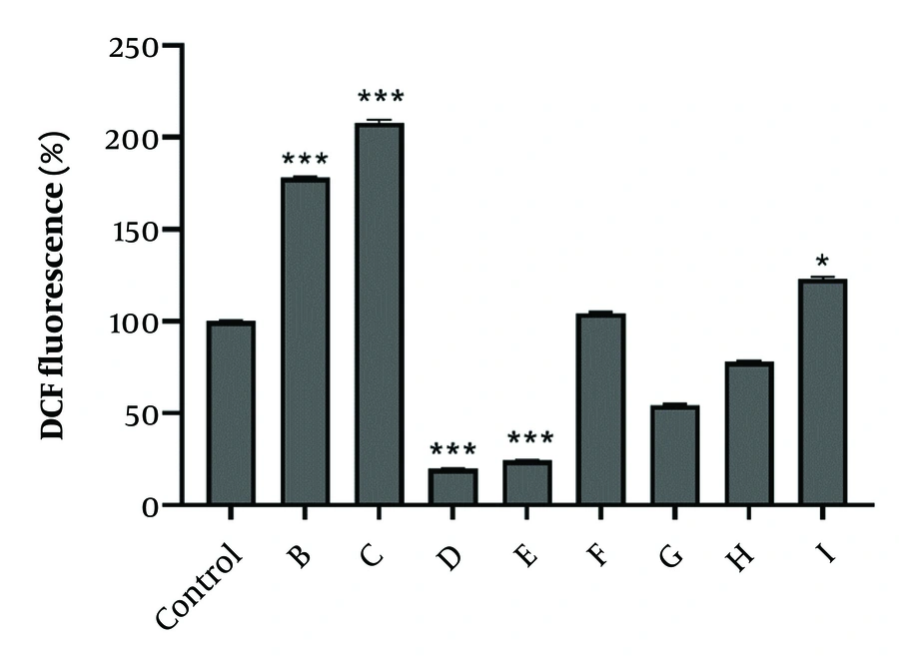
The effect of IC_50_ concentration of fractions obtained from total extracts of chloroform and ethyl acetate on the amount reactive oxygen species (ROS) detected by dichlorofluorescein diacetate (DCF). A2780 cells received fractions at the IC_50_ concentration. Data were expressed as the mean ± SEM, n = 3. * P < 0.05, and *** P < 0.001 indicate significant difference vs. control.

To measure oxidative stress induced by active fractions, the fluorescent dye DCF-DA was used to demonstrate ROS production. After 24-hour treatment of A2780 cells with the IC_50_ concentration of selected fractions, it was shown that fractions B, C, and I had a significant effect on ROS production and induction of oxidative stress. Interestingly, after 24-hour exposure to fractions D and E, ROS generation decreased in A2780 cells (Figure 4). 

## 5. Discussion

Medicinal plants have demonstrated usefulness in managing several diseases, including cancer. Several studies have shown that active compounds obtained from medicinal plants offer good therapeutic efficacy and lower side effects compared to synthetic products (18). In the present study, we aimed to evaluate the cytotoxic effects of different extracts and fractions obtained from the aerial parts of *S. lavandulifolia* on breast (MCF-7), lung (H1299), and ovarian (A2780) cancer cell lines. Identifying potent cytotoxic fractions is a crucial step toward discovering the active compounds of this plant.

According to our findings, chloroform and ethyl acetate extracts exhibited the most potent cytotoxic effects on the three human carcinoma cell lines. The IC_50_ values for chloroform and ethyl acetate extracts were 25 and 11 μg/mL in A2780, 28 and 14 μg/mL in MCF-7, and 29 and 31 μg/mL in H1299, respectively. In contrast, the water extract showed the lowest anticancer activity, with an IC_50_ value of > 300 μg/mL in all studied cell lines.

When examining the cytotoxic effects of fractions obtained from potent extracts, the A2780 human ovary carcinoma cell line was found to be more sensitive to increasing concentrations of fractions B-E from the chloroform extract and F-I from the ethyl acetate extract. Fraction D inhibited the growth of 50% of A2780 cells at a concentration of 11 μg/mL.

As chemotherapy drugs need to be selected for their potent cytotoxic effects on cancer cells, the cytotoxicity of the standard chemotherapeutic drug doxorubicin was evaluated in A2780 cells, identified as the most sensitive cell line. Doxorubicin demonstrated a significant cytotoxic effect with an IC_50_ value of 2.85 μg/mL. However, fractions C, D, F, and I were more potent than doxorubicin in the human ovarian carcinoma cell line.

Since targeting apoptotic pathways and inducing apoptosis in cancer cells is a critical approach in designing chemotherapeutic drugs, we further investigated the mode of cell death induced by potent fractions in the most sensitive cell line. Our mechanistic evaluation showed that fractions C, E, I, and F increased the activity of caspase-3, a key mediator of apoptosis whose activation is essential for initiating apoptosis in A2780 cells (19). Mitochondria play a principal role in initiating apoptosis through the intrinsic pathway. During apoptosis, a decrease in MMP leads to matrix condensation and cytochrome c release from the mitochondrial intermembrane space into the cytosol, causing activation of caspase-9. This caspase can activate executioner caspases, including caspase-3 (20).

The obtained results showed that fraction C decreased MMP in A2780 cells and subsequently increased caspase-9 activity, indicating the involvement of the intrinsic pathway of apoptosis by this fraction. Although fraction F decreased MMP in A2780 cells, caspase-9 activity did not change after exposure to this fraction, suggesting that this pathway is likely not involved in the observed cytotoxic effects of this fraction, and other mechanisms might account for its cytotoxic effect on A2780 cells.

Reactive oxygen species can induce different cell death mechanisms, such as apoptosis and necrosis (21). Regarding the apoptotic mechanism of cell death, ROS not only induce apoptosis through the intrinsic pathway but also have been shown to activate the extrinsic pathway of apoptosis (22). An increasing body of evidence shows that cancer cells possess higher ROS contents compared to normal non-cancerous cells and are thus prone to apoptotic death by drugs or therapies that induce oxidative stress, such as chemotherapy and radiotherapy (23). In our study, it was shown that fractions B, C, and I increased intracellular levels of ROS in A2780 cells. Since fractions C and I significantly increased the activation of caspase-3, this can be considered an apoptosis-inducing mechanism of these fractions.

Recent studies within the last five years have further elucidated the cytotoxic potential of the *Stachys* genus. For instance, Shakeri et al. identified diterpenoid quinones, such as 1-hydroxy-tanshinone IIA, from *S. parviflora*, demonstrating significant cytotoxicity against MCF-7, MDA-MB-231, and PC3 cell lines via apoptosis induction (24). Similarly, *S. viticina* essential oil, rich in endo-borneol and eucalyptol, exhibited strong antiproliferative effects against HeLa and Colo-205 cells (25). Phenolic compounds, including rosmarinic and chlorogenic acids, alongside flavonoids, were linked to cytotoxicity in *S. palustris* (26) and *S. byzantina* (27), while *S. pilifer* extracts showed anticancer activity (28). These findings underscore the presence of diverse cytotoxic phytochemicals across *Stachys* species, supporting their pharmacological relevance.

Despite extensive studies on the anticancer effects of other *Stachys* species, there are very few studies supporting the anticancer effect of *S. lavandulifolia* in human cancer cells. In a previous study, the cytotoxic effects of nine species of dichloromethane, methanol, and 80% methanol extracts of woundwort (*Stachys*) plants, including *S. lavandulifolia*, were evaluated on three human cancer cell lines (HL-60, MCF-7, and K-562) (29). The dichloromethane extract of *S. lavandulifolia* inhibited the growth of MCF-7 and HL-60 cells with IC_50_ values of 81.2 and 141 μg/mL, respectively. The 80% methanol extract of *S. lavandulifolia* was also active on MCF-7 and HL-60; however, its IC_50_ values were greater than 100 μg/mL in both. Interestingly, the methanol extract of *S. lavandulifolia* was not effective on the selected cancer cell lines.

In another study, the cytotoxic effects of CHCl_3_, EtOAc, and MeOH fractions of total extracts of species *S. laxa*, *S. trinervis*, *S. subaphylla*, and *S. turcomanica* were evaluated against colon carcinoma (HT-29), colorectal adenocarcinoma (CaCO-2), breast carcinoma (T47D), and fibroblast cells (NIH3T3). IC_50_ values showed that the growth and proliferation of HT-29 and T47D cells were most affected by chloroform and ethyl acetate fractions of *S. laxa* and *S. turcomanica* (30).

From a mechanistic point of view regarding responsible compounds, the cytotoxic/apoptotic activities of the related extracts and fractions could be due to the presence of various classes of phytochemicals. Research on the phytochemicals of the aerial parts of this plant found that its main components include D-germacrane (96.15%), thymol (14.44%), γ-Kadin (13.33%), α-pinene (7.80%), and trans-caryophyllene (6.91%). Moreover, this plant is an important source of monoterpenes and sesquiterpenes (31), and these compounds may be responsible for the cytotoxic activity of various *Stachys* species (17). Other studies suggested that aucubin and harpagide (iridoid glycosides) are probably responsible for the cytotoxic effects observed in these plants (32). The main compounds of essential oils are α-thujone (0.3 - 3.3%), α-pinene (37.3%), mirene (5.9% - 15.9%), β-phellandrene (11.9% - 37.9%), D-germacrane (4% - 11.3%), Δ-cadinen (11.6%), and 1,4-methano-1-H-indene (10.1%).

In a study conducted by the genetics and molecular biology department of Bingol in Ankara, Turkey, it was shown that oleic acid is one of the most abundant fatty acids in the genus *Stachys*. Other abundant fatty acids in this plant were linoleic acid and palmitic acid (33). In another study, the antiproliferative activity of extracts, flavonoids, and fatty acids isolated from the aerial parts of *S. byzantina* against Vero cells (the kidney of African immature monkeys), HeLa (human uterine carcinoma), and C6 (brain tumor in rats). The results demonstrated the presence of sixteen fatty acids in fractions. Moreover, hexane and ethyl acetate showed antiproliferative effects against all three cell lines (34).

Ma et al. separated an acidic polysaccharide fraction from the rhizomes of *S. floridana Schuttl. ex Benth *(SFPSA). This polysaccharide fraction was mainly composed of rhamnose, glucuronic acid, galacturonic acid, glucose, galactose, and arabinose. It has been shown that SFPSA had a potent cytotoxic effect on HT-29 colon carcinoma and was able to induce apoptosis through the activation of caspase-3 in HT-29 cells (34).

### 5.1. Conclusions

Based on the obtained results, fraction C is identified as the most effective fraction against the ovarian cancer cell line A2780. It can be proposed that, following the separation and identification of its active compounds, and subsequent in vivo studies on the A2780 cell line, this fraction may demonstrate significant anticancer effects. Additionally, there is potential for *S. lavandulifolia* to be utilized as a herbal drug with fewer side effects, either in combination with existing anticancer drugs or as a standalone treatment.

## Data Availability

The data that support the findings of this study are available on reasonable request from the corresponding author.
